# Assessing right ventricular deformation in hypertrophic cardiomyopathy patients with preserved right ventricular ejection fraction: a 3.0-T cardiovascular magnetic resonance study

**DOI:** 10.1038/s41598-020-58775-0

**Published:** 2020-02-06

**Authors:** Xiang Li, Ke Shi, Zhi-gang Yang, Ying-kun Guo, Shan Huang, Chun-chao Xia, Sen He, Zhen-lin Li, Chen Li, Yong He

**Affiliations:** 1 Department of Radiology, West China Hospital, Sichuan University, Chengdu, Sichuan China; 2 Department of Radiology, Key Laboratory of Obstetric & Gynecologic and Pediatric Diseases and Birth Defects of Ministry of Education, West China Second University Hospital, Sichuan University, Chengdu, Sichuan China; 3 Department of Cardiology, West China Hospital, Sichuan University, Chengdu, Sichuan China

**Keywords:** Cardiac hypertrophy, Imaging techniques

## Abstract

To assess the global and regional right ventricular (RV) deformation in hypertrophic cardiomyopathy (HCM) patients with preserved right ventricular ejection fraction (RVEF) using 3.0-T cardiovascular magnetic resonance tissue tracking (CMR-TT). Eighty-two HCM patients and 32 age- and sex-matched healthy controls were enrolled. HCM patients were divided into groups depending on the presence or absence of right ventricular hypertrophy (RVH), RV late gadolinium enhancement (RV-LGE), and left ventricular outflow tract obstruction (LVOTO), respectively. The RV global and apical longitudinal peak strain (LPS) in HCM patients with RVH were significantly lower than that in HCM patients without RVH and controls (P < 0.05). The global, apical and mid-ventricular LPS in HCM patients with RV-LGE were significantly lower than that in HCM patients without RV-LGE and controls (P < 0.05). Lower LPS was demonstrated in HCM patients without RV-LGE compared with controls in apical and mid-ventricular levels (P < 0.05). No significant difference was found regarding global and regional LPS in HCM patients with LVOTO compared without LVOTO (all P > 0.05). CMR-TT was able to detect subclinical RV myocardial deformation prior to RVEF impairment, which was more severe in the presence of RVH and RV-LGE.

## Introduction

Hypertrophic cardiomyopathy (HCM) is a primary and genetically transmitted cardiovascular disease with a prevalence of 1:500 in the general population. The condition is associated with a risk of adverse cardiac events such as severe arrhythmias, progressive heart failure, and sudden cardiac death^1–3^. It is also one of the most common causes of sudden cardiac death in young individuals and athletes^4^. In previous studies, increased right ventricular (RV) wall thickness and RV dysfunction in patients with HCM were shown to predict severe symptomatic HCM and poor prognosis^5,6^. RV ejection fraction (RVEF) has been typically used to assess the RV systolic function and has been shown to predict clinical outcomes of patients with cardiomyopathy^7^. However, the RVEF of patients with HCM tends to be within the “normal” range^8^. Currently, RV strain parameters are considered as more valuable markers of myocardium dysfunction than RVEF. RV strain parameters have been used for prognostic evaluation of multiple cardiovascular diseases and RV deformation in the healthy population^9–13^. The global and regional RV deformation in HCM patients with preserved RVEF is not well characterized^14–18^.

Cardiovascular magnetic resonance (CMR) is the standard reference modality for evaluation of RV structure and function^9,19^. CMR tissue tracking (TT) technology is a non-invasive method to assess myocardial deformation without the use of contrast media. Previous studies have applied CMR-TT technique with good intra- and inter-observer variability for determining the global and regional left ventricular (LV) deformation in the early stages of HCM^20,21^. However, to the best of our knowledge, information pertaining to RV strain in patients with HCM by CMR-TT is limited. Therefore, the aim of this study was to determine the global and regional RV deformation in patients with HCM with preserved RVEF by using CMR-TT.

## Results

### Patient characteristics

The basic characteristics of the study population were shown in Supplementary Table S1. In the HCM patient group, 29 patients were New York Heart Association (NYHA) class I, 48 were NYHA class II, 21 were NYHA class III, and 4 patients were NYHA class IV. Fifty-four (66%) had LVOTO; 65 (79%) had mitral valve regurgitation and 48 (59%) had mitral valve systolic anterior motion (SAM). There was no significant difference between HCM patients and controls with respect to RVEF (*P* = 0.087). Maximum RV free wall thickness in HCM patients was significantly greater than that in controls [median (25^th^, 75^th^ percentile): 4.56 (4.14–5.74) mm vs. 3.58 ± 0.56 mm, *P* < 0.05].

### RV myocardial deformation between HCM patients and controls

In patients with HCM, the global LPS [median (25^th^, 75^th^ percentile): −6.45% [−10.78%–(−3.49%)] vs. −9.54% ± 3.60%, *P* < 0.05], apical LPS [median (25^th^, 75^th^ percentile): −8.56% [−11.99%–(−5.97%)] vs. −13.14% [−15.65%–(−9.98%)], *P* < 0.05], and mid-ventricular LPS [median (25^th^, 75^th^ percentile): −5.83% [−10.91%–(−6.22%)] vs. −11.01% [−13.35%–(−5.94%)], *P* < 0.05] were significantly lower than those in controls (Table 1). There were no significant differences between HCM patients and controls with respect to global CPS or RPS (*P* > 0.05); however, the apical CPS, mid-ventricular CPS, and basal RPS in HCM patients were significantly lower as compared with controls (*P* < 0.05).

**Table 1:** 3D Peak strain parameters of right ventricle in control subjects and in HCM patients.

		Controls (n = 32)	HCM Patients (n = 82)	P
LPS	Global	−9.54 ± 3.60	−6.45 (−10.78–(−3.49))	0.007*
(%)	Apical	−13.14 (−15.65–(−9.98))	−8.56 (−11.99–(−5.97))	<0.001*
	Mid-ventricular	−11.01(−13.35–(−5.94))	−5.83 (−10.91−6.22)	0.001*
	Basal	−7.92 (−11.04–(−4.43))	−7.99 (−12.03–5.98)	0.847
CPS	Global	−3.12(−4.60–(−1.78))	0.23 (−4.33–7.92)	0.050
(%)	Apical	−8.36 (−12.16–(−6.50))	−6.50 (−9.35–3.98)	0.004*
	Mid-ventricular	−5.50 (−7.68–(−4.42))	−3.26 (−6.55–11.29)	0.001*
	Basal	12.41 (5.01−16.70)	7.33 (4.49–13.39)	0.075
RPS	Global	22.73 ± 8.24	18.39 (11.68−26.21)	0.120
(%)	Apical	11.28 (−1.31-15.55)	10.08 (4.44–15.46)	0.685
	Mid-ventricular	20.60 ± 10.29	19.82 (8.39–28.52)	0.677
	Basal	42.89 (31.05–50.34)	30.65 (21.60–48.61)	0.035*

Notes: Data are expressed as mean ± SD or median (25^th^, 75^th^ percentile); *P < 0.05 versus controls; Man-Whitney U test was performed to evaluate the differences in continuous variables between two groups. HCM, hypertrophic cardiomyopathy; 3D, three dimensional; LPS, longitudinal peak strain; CPS, circumferential peak strain; RPS, radial peak strain.

### Myocardial deformation based on RVH, RV-LGE, and LVOTO

#### RVH

**Figure 1:**
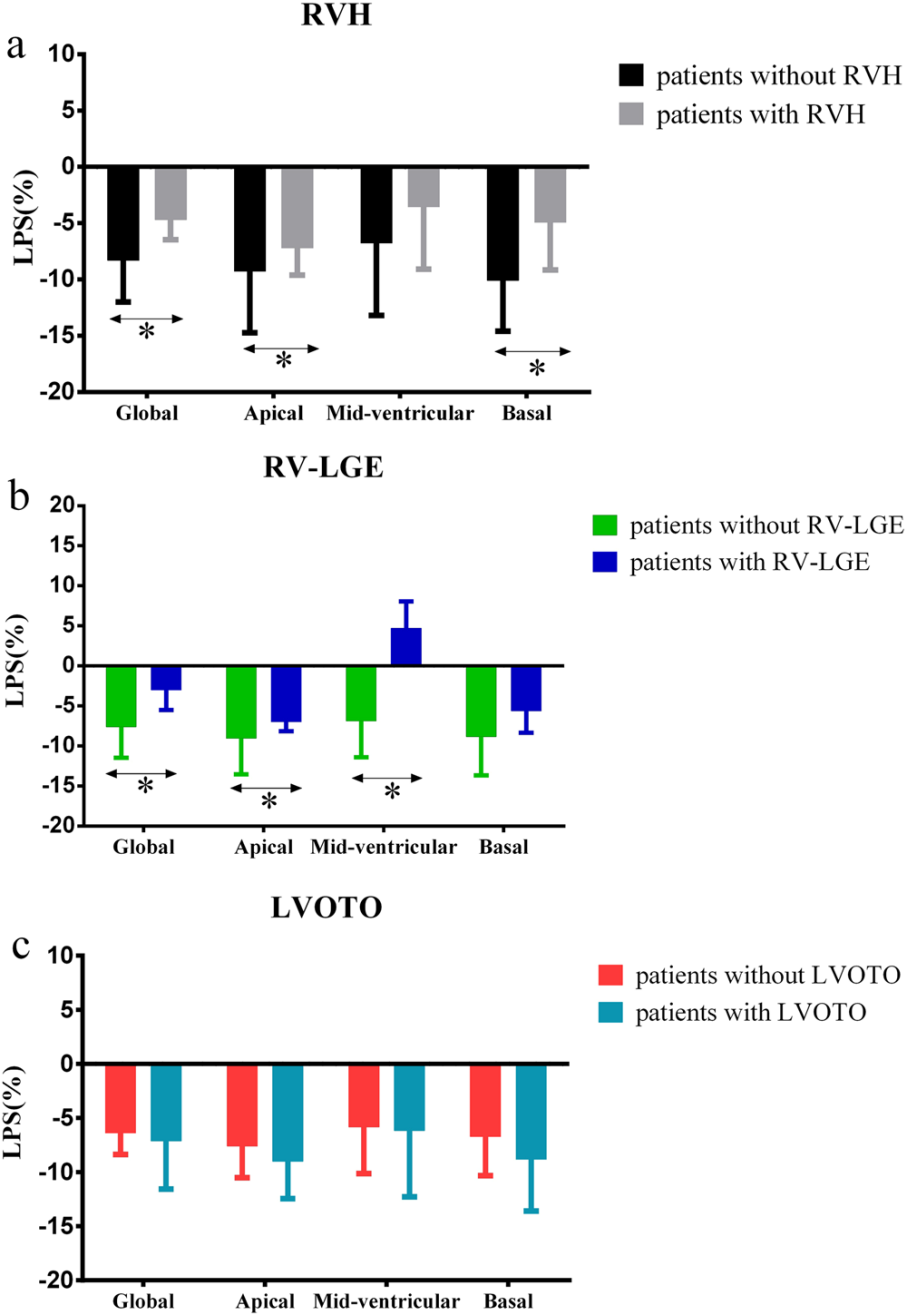
Bar graphs for comparison of global and regional (apical, mid-ventricular and basal) peak strain in longitudinal direction in HCM patients based on RVH (**a**), RV-LGE (**b**), and LVOTO. (**c**) The Kruskal-Wallis test was used to evaluate the differences in continuous variables between two groups. Bar represents the median; horizontal box line represents the interquartile range; asterisks show significant differences (*P* < 0.05) between two groups. HCM, hypertrophic cardiomyopathy; RVH, right ventricular hypertrophy; RV-LGE, late gadolinium enhancement in right ventricle; LVOTO, Left ventricular outflow tract obstruction.

Thirty-two and 50 HCM patients presented with and without RVH, respectively. The maximum RVFWT of HCM patients presented with RVH was obvious higher than those in HCM patients without RVH [median (25^th^, 75^th^ percentile): 6.12 (5.66–8.03) mm vs. 4.26 (3.17–4.51) mm, *P* < 0.05]. Of the HCM patients with RVH, 19(59%) had apical RVH; 18(56%) had mid-ventricular RVH and 5(16%) had basal RVH. The global RV longitudinal peak strain (LPS) [median (25^th^, 75^th^ percentile): −4.70% (−6.47%–1.74%) vs. −8.31% [−12.00%–(−5.58%)], −4.70% (−6.47%–1.74%) vs. −9.54% ± 3.60%, *P* < 0.05, respectively] and apical LPS [median (25^th^, 75^th^ percentile): −7.21% [−9.62%–(−3.74%)] vs. −9.26% [−14.74%–(−6.48%)], −7.21% [−9.62%–(−3.74%)] vs. −13.14% [−15.65%–(−9.98%)], *P* < 0.05, respectively] in HCM patients with RVH were significantly lower than those in HCM patients without RVH and controls. Patients with RVH showed a tendency for lower mid-ventricular LPS as compared to controls (*P* < 0.05). The basal RV LPS in HCM patients with RVH was significantly lower than that in HCM patients without RVH (*P* < 0.05). There were no differences of the global and regional CPS and RPS between HCM patients with RVH and HCM patients without RVH (*P* > 0.05) (supplementary Table S2, Fig. 1a).

#### RV-LGE

Thirteen and 69 HCM patients presented with and without RV-LGE, respectively. As for the location of RV-LGE in HCM patients, 5(38%) had the apical RV-LGE, 10 (77%) had middle ventricular RV-LGE, while only 1(8%) showed at the RV basal region. The global RV LPS [median (25^th^, 75^th^ percentile): −2.99% (−5.50%–6.80%) vs. −7.60% [−11.48%–(−4.88%)], −2.99% (−5.50%–6.80%) vs. −9.54% ± 3.60%, *P* < 0.05, respectively], apical LPS [median (25^th^, 75^th^ percentile): −6.97% (−8.15%–1.98%) vs. −9.05% [−13.53%–(−6.23%)], −6.97% (−8.15%–1.98%) vs. −13.14% [−15.65%–(−9.98%)], *P* < 0.05, respectively] and mid-ventricular LPS [median (25^th^, 75^th^ percentile): 4.70% (−4.40%–8.07%) vs. −6.86% (−11.43%–5.88%), 4.70% (−4.40%–8.07%) vs. −11.01% [−13.35%–(−5.94%)], *P* < 0.05, respectively] in HCM patients with RV-LGE were significantly reduced compared with HCM patients without RV-LGE and controls. There was no significant difference between HCM patients with and without RV-LGE with respect to global and regional CPS as well as RPS (*P* > 0.05) (Supplementary Table S2, Fig. 1b).

#### LVOTO

Fifty-four HCM patients presented with LVOTO and 28 presented without LVOTO. There were no significant differences between the two groups of HCM patients with respect to global and regional LPS, CPS, or RPS (*P* > 0.05). The apical and mid-ventricular CPS and LPS of HCM patients with LVOTO were significantly lower than those in controls (*P* < 0.05). In addition, the LPS values of HCM patients without LVOTO at the apical and mid-ventricular levels were decreased compared with controls (*P* < 0.05) (Supplementary Table S2, Fig. 1c).

### Reproducibility of RV strain

Measurements of global RV strain in the longitudinal direction exhibited good intra- [intraclass correlation coefficient (ICC) = 0.973; 95% confidence interval (CI), 0.932–0.989] and inter-observer (ICC = 0.971; 95% CI, 0.927–0.988) reproducibility. Global CPS showed moderate intra- (ICC = 0.914; 95% CI, 0.782–0.966) and inter-observer (ICC = 0.855; 95% CI, 0.639–0.942) agreement. The inter-observer ICC of global RPS was 0.864, while the intra-observer ICC was 0.875 (Table 2).

**Table 2:** Intra- and Interobservers variability of CMR tissue tracking by the ICC Analysis.

	Intra-observer variability		Inter-observer variability	
	ICC	95% CI	ICC	95% CI
GLPS (%)	0.973	0.932–0.989	0.971	0.927–0.988
GCPS (%)	0.914	0.782–0.966	0.855	0.639–0.942
GRPS (%)	0.864	0.653–0.946	0.875	0.684–0.951

Note: ICC, Intraclass correlation coefficient; 95% CI, 95% Confidence interval. CMR, cardiovascular magnetic resonance; GLPS, global longitudinal peak strain; GCPS, global circumferential peak strain; GRPS, global radial peak strain.

## Discussion

The main findings of our study were as follows: 1) Global and regional RV LPS strain in HCM patients with RVH were lower than those in HCM patients without RVH. 2) Global and regional RV LPS in individuals with RV-LGE were lower than those in controls. 3) There were no significant differences with respect to RV strain between HCM patients with LVOTO and those without LVOTO. CMR-TT has the capability to quantitatively assess the global and regional RV myocardial strain in HCM patients with preserved RVEF with high reproducibility in longitudinal direction.

RV strain parameters are typically regarded as markers of myocardial deformation and can be measured by many CMR myocardial tracking technologies. CMR-TT makes use of the near incompressibility of the myocardium and allows for the assessment of myocardial deformation by tracking both endocardial and epicardial boundary voxels^20^. It precludes the use of complex post-processing procedure in CMR tagging technology^22^; in addition, the intra-observer reproducibility of CMR-TT strain analysis is independent of magnetic field intensity^23^. In this study, we observed good inter- and intra-observer reproducibility for global LPS and CPS in HCM patients, which is consistent with previous studies^21^.

RV wall thickness has been shown to be a common structural RV abnormality in HCM patients^5,8,18^. In this study, we found increased maximum RVFWT in all HCM patients, which is consistent with previous studies^14,18^. This phenomenon may be attributable to a combination of genetic mutation that encodes sarcomere proteins, variation of afterload, or ventricular interdependence; however, further studies are required to elucidate the main trigger mechanism^24^. Moreover, previous studies demonstrated that RV deformation is important to assess the outcomes of patients with HCM^14,25^. Most previous studies have focused on LV changes in HCM patients, while few studies have characterized RV deformation in HCM patients using CMR. We found that global and regional RV LPS were impaired and RV CPS was regionally decreased in all HCM patients with preserved RVEF. Further, global RV LPS in HCM patients with preserved RVH was lower than that in patients without RVH and control subjects. In our study, impaired regional myocardial deformation mainly occurred at the apical and mid-ventricular levels and the basal myocardial deformation was not decreased compared with controls. This was corresponding to the location of RVH in HCM patients we found. Previous study about the analysis of LV strain has shown that the myocardial deformation of hypertrophic region was more impaired than nonhypertrophic region^21^. The mode of our group and found were similar to their study. We conjectured that the hypertrophy and disarray of myocardial cell in RV may have caused changes in RV deformation and kinetic feature.

The presence of LGE in LV has always been considered as indicative of myocardial fibrosis and a prognostic marker in HCM patients^26^. In our study, RV-LGE was also observed in HCM patients, which suggested that the RV of HCM patients may be affected by myocardial fibrosis. HCM patients with RV-LGE had lower values of global and regional LPS compared with patients without RV-LGE and controls; these findings also supported that fibrosis might lead to myocardial dysfunction. In addition, the location of RV-LGE was similar to that of RVH, i.e., largely confined to the intersection areas of two ventricles at the apical and mid-ventricular levels; this suggested that the pathologic changes in RV were broadly similar to those in the LV^27^. The presence of RV-LGE may have some clinical value in the prognostic assessment of HCM patients; however, further studies are required to obtain more definitive evidence.

Increase in the left ventricular out flow gradient is an important marker of development of LVOTO in patients with HCM^2^. Previous studies have shown that patients with HCM who have LVOTO are at a higher risk of sudden cardiac death or progression to severe congestive symptoms^28,29^. Studies have also shown that inter-ventricular septum is shared by two chambers and that the LV motion may affect RV motion by direct mechanical interaction^6^. However, we observed no significant differences in RV strains between HCM patients with LVOTO and those without LVOTO, which is consistent with previous studies^14^. These findings suggest that LV hemodynamic changes induced by LVOTO may have little impact on RV deformation in patients with preserved RVEF.

Our study has the following limitations. Firstly, this was a single-center retrospective study; multicenter study with a larger cohort is required to confirm our findings. Secondly, the normal reference values of global and regional 3D RV strain by CMR TT are not well determined, but our results were similar to our team’s previous article^30^. The quantitative data of myocardial strain using different software might be different, thus more work is required to determine the normal reference values of global and regional 3D RV strain. Finally, genetic-test and follow-up data which unavailable for this study should be acquired to assess the clinical relevance of RV strain parameters as prognostic markers in HCM patients in our further studies.

To summarize, in this study, decreased global and regional RV myocardial strain were detected prior to the reduction in RVEF, which suggests subclinical impairment of RV strain in HCM patients compared with controls. Moreover, impaired RV myocardial deformation was more obvious in the presence of RVH and RV-LGE. The location of impaired regional RV strain was mainly confined to the apical and mid-ventricular region, which was consistent with the locations of RVH and RV-LGE. Although the two ventricular chambers show interdependence and LVOTO can lead to LV hemodynamic abnormality, LVOTO may play a limited role in RV deformation in HCM patients with preserved RVEF.

## Methods

### Patient population

In this retrospective study, we recruited 169 patients who were diagnosed with HCM (according to the criteria recommended by the ACCF/AHA and ESC guidelines) between January 2010 and January 2018 at our institution^2,3^. Preserved RVEF was defined as RVEF ≥ 45% in this study^18^. Patients who presented with any of the following conditions were excluded: hypertension (n = 53), severe coronary artery disease (n = 11), previous treatment such as myomectomy or ablation (n = 7), or poor or false CMR images (n = 18). Finally, 82 patients with HCM (average age 46 ± 16 years; 49 men and 33 women) with preserved RVEF were enrolled. Patients were divided into groups depending on the presence (n = 32) or absence (n = 50) of right ventricular hypertrophy (RVH), on presence (n = 13) or absence (n = 69) of late gadolinium enhancement in RV (RV-LGE), and on presence (n = 54) or absence (n = 28) of left ventricular outflow tract obstruction (LVOTO). RVH was defined as RV maximum thickness at the end of diastole more than 5 mm as measured by CMR^31,32^. LVOTO was defined as peak LV outflow tract pressure gradient ≥30 mmHg, as measured by echocardiography^2,3^.

In this study, 32 age- and sex-matched healthy individuals were enrolled as the control group (mean age, 51 ± 13 years; 19 men and 13 women). The exclusion criteria for controls were: chronic disease, family history of cardiovascular disease, hypertension (blood pressure > 140/90 mmHg), or severe arrhythmias. This study was approved by the Institutional Ethics Review Board of West China Hospital and performed in accordance with the ethical guidelines of the Declaration of Helsinki (2013 EDITION)^33^. Written informed consent was obtained from all patients and controls.

### CMR acquisition protocol

The CMR images were acquired using a 3.0-T CMR scanner (Skyra; Siemens Medical Solutions, Erlangen, Germany) with body phased array coil. All subjects assumed the supine position and remained stable during the entire process. Electrocardiogram (ECG) and breathing were monitored by the manufacturer’s standard ECG-triggering device and the end-expiratory breath-holding technique. All images were obtained during the breath-holding period. Vertical 2-chamber long axis, horizontal 4-chamber long axis, and 8-12 contiguous short-axis cine series from the base to the apex level were acquired using a Turbo FLASH sequence. The typical sequence parameters were: repetition time (TR) = 47.88 ms; echo time (TE) = 1.51 ms; flip angle = 40°; field of view (FOV) = 265 × 340 mm^2^; slice thickness = 8.0 mm; matrix size = 200 × 256. Fifteen minutes after intravenous injection of 0.2 ml/kg gadolinium chelate contrast agent (Gadobenate dimeglumine (MultiHance), 0.5 mmol/ml; Bracco, Milan, Italy), late gadolinium enhancement (LGE) images were acquired using the phase-sensitive inversion recovery (PSIR) sequence (TR = 700 ms; TE = 1.18 ms; flip angle = 40°; FOV = 275 × 400 mm^2^; slice thickness = 8.0 mm; matrix size = 184 × 256).

### CMR data analysis

**Figure 2:**
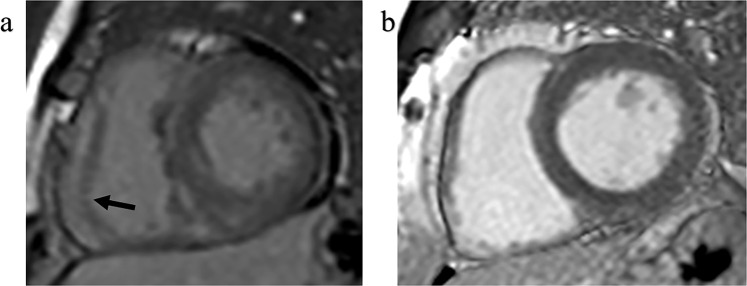
Late gadolinium enhancement area in RV free wall of HCM patients (**a**, the arrows) compared with the normal reference in RV free wall of a normal individual. (**b**) HCM, hypertrophic cardiomyopathy; RV, right ventricular.

All CMR data were analyzed off-line by an experienced radiologist (three years of experience in CMR) using commercially available software (cvi42; Circle Cardiovascular Imaging, Calgary, Canada). In each short-axial cine dataset, we delineated the optimal endocardial and epicardial borders of both ventricles at the end-diastolic and end-systolic phase and defined the insertion of RV and LV as the reference point. The ventricular extent was defined as extending from the bicuspid or tricuspid valve to the apex in the horizontal 4-chamber long axis view. Papillary muscles, and moderator bands were diligently excluded. Global cardiac structural parameters, including biventricular ejection fraction (EF), end-diastolic volume (EDV), end-systolic volume (ESV), stroke volume (SV) and diastolic cardiac mass, and the maximum LV end-diastolic thickness (EDTH) were generated automatically by the cvi42 short−3D module of the software. Maximal RV free wall thickness (RVFWT) was measured manually from images of end-diastolic phase. The RV-LGE was a qualitative parameter and determined by two experienced radiologists in a blinded manner (Fig. 2).

**Figure 3:**
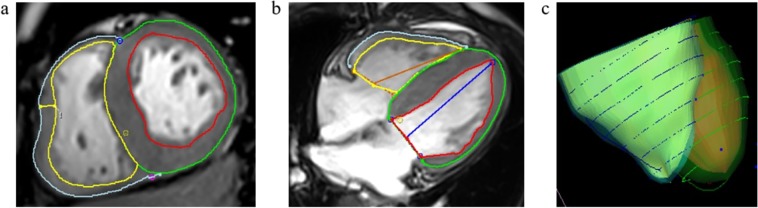
The establishment process of CMR tissue tracking model in a patient with HCM. The optimal endocardial (yellow and red curves) and epicardial borders (the cyan and green curves) of the two ventricles were outlined at the end-diastolic phase of loaded short-axis (**a**) and long-axis four-chamber (**b**) cine datasets and the insertion of RV and LV as reference points (a, the cyan and pink dots) were defined. The maximum RVFWT was measured (a, the yellow line). The ventricular extent was defined as extending from the bicuspid or tricuspid valve to the apex in horizontal 4-chamber long axis view (**b**). Three-dimensional (3D) tracking model (**c**) was automatically constructed by the software after the end-systole phase was defined. CMR, cardiovascular magnetic resonance; LV, left ventricular; RVFWT, thicken of right ventricular free wall; HCM and RV as Fig. 2.

**Figure 4:**
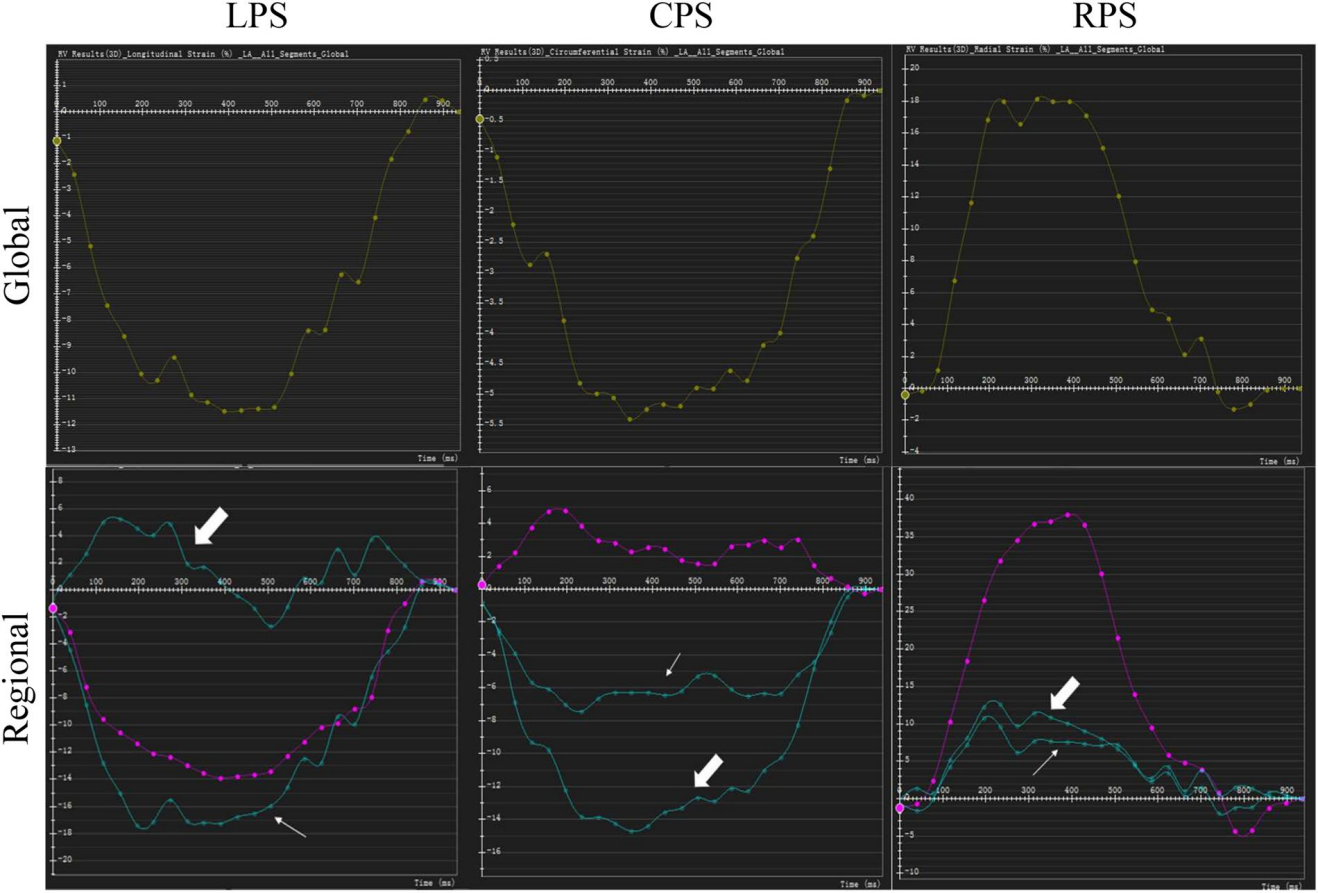
Values of RV free wall myocardium strain in multiple directions in HCM patient with RVH, including the longitudinal, circumferential and radial peak strain (LPS, CPS, RPS) of global (upper row) and regional (lower row) RV free wall. Red curve shows the values at the basal position; the thick arrow indicates the mid-ventricular level; the thin arrow indicates the ventricular apex. RVH, right ventricular hypertrophy; HCM and RV as Fig. 2.

The tissue tracking module of our software was used to obtain RV strain data. In a set of loaded long-axis four-chamber and short-axis cine datasets, the optimal RV endo-and epicardial borders at the end-diastolic phase were drawn and considered as the reference position of other cine datasets. The software utilized the near incompressibility of myocardium to automated strain analysis and construct a three-dimensional (3D) deformable myocardial model (Fig. 3). The accuracy and good quality of tracking were verified by visual check and adjustments of the observer. Subsequently, 3D global and regional (basal, mid-ventricular, and apical level) RV strain variables including longitudinal, circumferential, and radial peak strain (LPS, CPS, RPS) were derived (Fig. 4). Peak strain was defined as the absolute value of the maximum strain measured throughout the cardiac cycle. The negative sign reflects the shortening of myocardium, and vice versa^34^.

### Reproducibility

Intra-observer variability was assessed by comparison of measurement of the same observer in randomly selected 20 individuals. The time interval between measurements made by the same observer was 2 weeks. Inter-observer variability was assessed by two independent experienced and double-blinded observers for the same individuals. All the CMR readers had three years of experience in diagnosing CMR images.

### Statistical analysis

Statistical analysis was performed using IBM SPSS (v. 21.0, Armonk, NY) and MedCalc (Mariakerke, Belgium, v.9.2.00). Continuous variables are expressed as mean ± standard deviation (SD) or as median (25^th^, 75^th^ percentile). Categorical data are presented as frequencies and percentages. Man-Whitney U test or Student’s *t* test was used to compare biventricular function parameters between HCM patients and controls. Fisher exact test was used to assess differences between male and female patients; differences of global and regional myocardial peak strains between groups of patients and controls were assessed with the Kruskal–Wallis test. Intra- and inter-observer variability with respect to RV peak strain was assessed using intraclass correlation coefficient (ICC). Two-tailed *P* values less than 0.05 were considered statistically significant.

## Supplementary information

Supplementary information.

## Data Availability

All data used during this study will be available from the corresponding author if the request is rational.
