# Supplementary material for: Assessing right ventricular deformation in hypertrophic cardiomyopathy patients with preserved right ventricular ejection fraction: a 3.0-T cardiovascular magnetic resonance study
Source: Sci Rep. 2020 Feb 6;10:1967. doi: 10.1038/s41598-020-58775-0 (PMC7004999; doi:10.1038/s41598-020-58775-0)
Supplement: Supplementary file 1 — Supplementary information. [file 41598_2020_58775_MOESM1_ESM.pdf]

Assessing right ventricular deformation in hypertrophic cardiomyopathy patients with preserved right ventricular ejection fraction: a 3.0-T cardiovascular magnetic resonance study

Xiang Li[1#]; Ke Shi[1#]; Zhi-gang Yang[1*]; Ying-kun Guo[2*]; Shan Huang[1]; Chun-chao Xia[1]; Sen He[3]; Zhen-lin Li[1]; Chen Li[3]; Yong He[3]

1. Department of Radiology, West China Hospital, Sichuan University, Chengdu, Sichuan, China.
2. Department of Radiology, Key Laboratory of Obstetric & Gynecologic and Pediatric Diseases and Birth Defects of Ministry of Education, West China Second University Hospital, Sichuan University, Chengdu, Sichuan, China.
3. Department of Cardiology, West China Hospital, Sichuan University, Chengdu, Sichuan, China.

[#] Xiang Li and Ke Shi contributed equally to this work.

* Zhi-gang Yang and Ying-kun Guo contributed equally to this work.

Table S1. Baseline of the controls and HCM patients

	Controls (n =32)	HCM patients (n =82)	*P*
**Age (year)**	51 ± 13	46 ± 16	0.186
**Sex (male; n, %)**	19 (60%)	49 (60%)	0.438
**Angina (n, %)**	N/A	28 (35.96%)	-
**Drinking (n, %)**	N/A	17 (21%)	-
**Smoking (n, %)**	N/A	25(30%)	-
**HR (beats/min)**	71.91 ± 8.14	72.00(63.00−80.00)	-
**NYHA classification**	N/A	29/48/21/4	-

**(I/II/III/IV)**			
**Echocardiographic parameters**			
**Mitral valve regurgitation (n, %) (mild/moderate severe)**	N/A	65(79%) (44/19/2)	-
**Tricuspid valve regurgitation (n, %) (mild/moderate severe)**	N/A	21 (26%) (21/0/0)	-
**Aortic valve regurgitation (n, %) (mild/moderate severe)**	N/A	14 (17%) (14/0/0)	-
**SAM (n, %)**	N/A	48 (59%)	-
**LVOTO (n, %)**	N/A	54 (66%)	-
**CMR parameters**			
**RVEDV (ml)**	108.03(95.13−134.42)	96.22 ± 25.29	0.002*
**RVESV (ml)**	51.93 ± 17.08	40.06(28.30−47.87)	<0.001*
**RVSV (ml)**	59.93(51.61−78.15)	54.89(43.50−65.39)	0.023*
**RVEF (%)**	56.31 ± 7.09	58.59(53.40−63.76)	0.087
**RV mass (g)**	23.30 ± 7.47	26.08(22.00−32.75)	0.007*
**Max RVFWT (mm)**	3.58 ± 0.56	4.56(4.14−5.74)	<0.001*
**LVEDV (ml)**	124.19 ±27.23	142.40 ± 34.81	0.009*
**LVESV (ml)**	52.08 ± 13.88	51.01(37.70−63.25)	0.614
**LVSV (ml)**	67.77(58.01−87.85)	89.68 ± 21.62	<0.001*
**LVEF (%)**	57.50 ± 4.06	63.55 ± 8.21	<0.001*
**LV mass (g)**	68.27± 20.20	126.25(103.35−165.29)	<0.001*
**Max LV EDTH (mm)**	9.62 ± 1.68	18.69(16.13–22.27)	<0.001*

Notes: Data are expressed as mean ± SD or median (interquartile range, 25% to 75%) or absolute numbers (percentages); Man-Whitney U test or Student's *t* test was used to compare biventricular function parameters between HCM patients and controls. Fisher exact test was used to assess differences between male and female patients. *$P < 0.05$

versus controls. HCM, hypertrophic cardiomyopathy; HR, heart rate; NYHA, New York Heart Association; SAM, systolic anterior movement; LVOTO, left ventricular outflow tract obstruction; CMR, cardiovascular magnetic resonance; RV, right ventricular; LV, left ventricular; EDV, end diastolic volume; ESV, end systolic volume; SV, stroke volume; EF, ejection fraction; Max RVFWT, maximum thicken of right ventricular free wall. Max LV EDTH, maximum end-diastolic thickness of left ventricle.

Table S2. Global and regional RV strain in HCM patients based on RVH, RV-LGE, and LVOTO.

		RVH		RVLGE		LVOTO	
		Patients without RVH (n=50)	Patients with RVH (n=32)	patients without RV-LGE (n=69)	patients with RV-LGE (n=13)	patients without LVOTO (n=28)	patients with LVOTO (n=54)
**LPS**	Global	−8.31	−4.70 *†	−7.60	−2.99 *†	−6.39 *	−7.12
**(%)**		(−12.00−(−5.58))	(−6.47−1.74))	(−11.48−(−4.88))	(−5.50−6.80)	(−8.36−(−3.09))	(−11.56−(−3.63))
	Apical	−9.26 *	−7.21 *†	−9.05 *	−6.97 *†	−7.59 *	−9.00 *
		(−14.74−(−6.48))	(−9.62−(−3.74))	(−13.53−(−6.23))	(−8.15−1.98)	(−10.53−(−3.09))	(−12.45−(−5.99))
	Mid-ventricular	−6.76	−3.56 *	−6.86 *	4.70 *†	−5.83 *	−6.16 *
		(−13.19−5.89)	(−9.08−6.90)	(−11.43−5.88)	(−4.40−8.07)	(−10.13−4.38)	(−12.29−9.01)
	Basal	−10.10	−4.93†	−8.83	−5.61	−6.72	−8.84
		(−14.60−(−6.14))	(−9.14−7.88)	(−13.66−4.71)	(−8.34−11.38)	(−10.32−2.21)	(−13.59− 6.53)
**CPS**	Global	0.23	0.36	−1.84 *	3.65 *	−3.42	3.73
**(%)**		(−4.55−8.28)	(−4.19−7.09)	(−4.45−8.09)	(−4.18−7.70)	(−4.70−7.27)	(−4.26−8.00)
	Apical	−6.69	−5.86 *	−6.54 *	−5.88	−6.43	−6.50 *

		(−9.51−(−2.06))	(−8.69−6.41)	(−9.39−(−3.05))	(−8.16−8.97)	(−9.25−(−3.47))	(−9.38−5.34)
	Mid-ventricular	−2.36 *	−4.21	−2.50	−3.92	−4.10	−2.48 *
		(−5.96−9.86)	(−7.68−14.11)	(−6.98−11.74)	(−6.08−10.98)	(−7.80−8.21)	(−6.27−13.64)
	Basal	8.08	6.28	7.82	7.11	6.08	8.08
		(4.47−14.00)	(4.21−12.20)	(4.68−13.04)	(−0.08−15.40)	(4.30−9.08)	(4.45−14.62)
**RPS**	Global	18.76	17.89	20.20	14.84	16.08	18.93
**(%)**		(11.27−26.73)	(12.08−25.98)	(11.86−26.29)	(11.13− 23.60)	(11.44−26.10)	(11.68−26.73)
	Apical	8.96	12.34	9.65	10.92	11.15	9.72
		(−4.04−12.31)	(7.57−19.79)	(4.42−15.23)	(5.29−20.99)	(4.52−14.90)	(3.82−15.73)
	Mid-ventricular	18.97	20.51	20.05	15.78	18.57	20.03
		(7.78−28.52)	(10.51−29.05)	(8.38−28.53)	(8.52− 37.32)	(7.52− 28.04)	(10.96−29.61)
	Basal	31.52	28.09	31.65	26.06 *	28.09	31.06
		(22.18−53.08)	(20.70−43.16)	(21.91−49.01)	(17.50− 35.22)	(22.97−51.74)	(20.39−47.71)

Notes: Data are expressed as median (25th, 75th percentile); **P* < .05 versus controls; †*P* < .05 versus patients. The Kruskal-Wallis rank test was performed to evaluate the differences in continuous variables between groups of patients and control. HCM, hypertrophic cardiomyopathy; RVH,

right ventricular hypertrophy; RV-LGE, late gadolinium enhancement in right ventricle; LVOTO, Left ventricular outflow tract obstruction; LPS, longitudinal peak strain; CPS, circumferential peak strain; RPS, radial peak strain.
